# Thoracic Outlet Syndrome in Sport: A Systematic Review

**DOI:** 10.3389/fphys.2022.838014

**Published:** 2022-06-08

**Authors:** Thomas Garraud, Germain Pomares, Pauline Daley, Pierre Menu, Marc Dauty, Alban Fouasson-Chailloux

**Affiliations:** ^1^ Hôpital Privé du Confluent, Rhumatologie, Nantes, France; ^2^ Service de Médecine du Sport, CHU Nantes, Nantes, France; ^3^ Institut Européen de la Main, Luxembourg. Luxembourg; ^4^ Medical Training Center, Hopital Kirchberg, Luxembourg. Luxembourg; ^5^ CHU Nantes, Service de Médecine Physique et Réadaptation Locomotrice et Respiratoire, Nantes, France; ^6^ Inserm, UMR 1229, RMeS, Regenerative Medicine and Skeleton, Université de Nantes, ONIRIS, Nantes, France; ^7^ IRMS, Institut Régional de Médecine du Sport, Nantes, France

**Keywords:** thoracic outlet syndrome, athletes, sport, management, neurogenic

## Abstract

Thoracic outlet syndrome (TOS) is a rare and heterogeneous syndrome secondary to a compression of the neurovascular bundle in the thoracic outlet area. Muscle hypertrophy is recognized to induce vascular or neurogenic compression, especially in sports involving upper-arm solicitation. Athletes represent a distinctive population because of a specific management due to an ambitious objective, which is returning to high-level competition. We evaluated the scientific literature available for the management of TOS in athletes. Article research extended to March 2021 without other restriction concerning the date of articles publication. The search was performed independently by two assessors. A first preselection based on the article titles was produced, regarding their availability in English or French and a second preselection was produced after reading the abstracts. In case of doubt, a third assessor’s advice was asked. Case reports were selected only if the sport involved was documented, as well as the level of practice. Cohorts were included if data about the number and the sport level of athletes were detailed. Seventy-eight articles were selected including 40 case reports, 10 clinical studies and 28 reviews of literature. Baseball pitchers seem to be highly at risk of developing a TOS. The surgical management appears particularly frequent in this specific population. The prognosis of TOS in athletes seems to be better than in the general population, possibly due to their better physical condition and their younger age. Some studies showed interesting and encouraging results concerning return to previous sport level. Literature shows a strong link between TOS and certain sports. Unfortunately, this syndrome still lacks rigorous diagnostic criteria and management guidelines for athletes.

## Introduction

Thoracic outlet syndrome is a heterogeneous entity defined by all the symptoms caused by vascular or neurologic compression in the anatomical region of the thoracic outlet. This anatomical area is subdivided in three potential compression sites for the neurovascular bundle (Jones et al., 2019):

- The interscalene triangle (compression of the brachial plexus or the sub-clavian artery) delimited by the anterior scalene muscle, the first rib at the bottom and the middle scalene muscle.
- The costo-clavicular space containing the brachial plexus and the sub-clavian vessels, delimited postero-inferiorly by the first rib, anteriorly by the subclavian muscle and the inferior aspect of the clavicle.
- The sub-coracoid space containing the axillary vessels and the brachial plexus, delimited anteriorly by the tendon of pectorals minor inserting onto the coracoid process (Hertslet and Keith, 1896; Craig and Knepper, 1937; Peet et al., 1956; Jones et al., 2019).

Peet et al. (1956) proposed the term of Thoracic Outlet Syndrome (TOS) in 1956, in order to summarize all symptoms caused by compression of the neurovascular bundle in this anatomical area. Diseases were previously defined by the finding or not of a predisposal anatomical factor: first rib syndrome or scalenus anticus syndrome for instance (Hertslet and Keith, 1896; Craig and Knepper, 1937).

Three forms of TOS syndrome are described. The N-TOS for Neurological TOS involves the compression of the brachial plexus. It is the most frequent form concerning about 90%–95% of total TOS cases recorded. The Venous TOS or V-TOS concerns about 5%–10% of the total cases. This specific form can lead to thrombosis of upper arm veins in 10%–20% of cases and sometimes leads to pulmonary embolism (Farrar et al., 2014). This acute complication is called Paget-Schroetter syndrome (PSS). The arterial TOS (A-TOS) is rare and involves blood flow restriction or occlusion of the axillary or sub-clavian artery and leads to upper limb ischemia (Jones et al., 2019). It is also usual to assess the presence or not of a predisposing anatomical factor during the diagnostic process (Watson et al., 2010; Freischlag and Orion, 2014; Levine and Rigby, 2018). For instance, cervical rib is found in about 1.1% of the general population versus 29.8% in TOS (Henry et al., 2018). It is also possible to present N-TOS associated vascular TOS symptoms. In a pediatric population, up to 28% of the patients with V-TOS had also N-TOS (Matos et al., 2018).

Diagnosis of TOS is challenging, as the wide panel of symptoms described is nonspecific especially in N-TOS. Therefore there is a lack of precise diagnostic criteria (Twaij et al., 2013; Povlsen et al., 2014; Masocatto et al., 2019). Most of the time TOS is diagnosed after exclusion of other pathologies (Freischlag and Orion, 2014). The diagnosis of A-TOS is essentially based on ischemic symptoms of the upper limb with objective arterial damage with the exclusion of asymptomatic hemodynamic changes and pulse obliteration (Illig et al., 2016). V-TOS can be suspected in case of arm swelling with discoloration and heaviness, and abnormal visibility of the chest venous collaterals (Illig et al., 2016). N-TOS diagnosis is more difficult with multiple symptoms, usually including pain, arm weakness, and strength deficit (Fouasson-Chailloux et al., 2021a; Daley et al., 2021), but the CORE-TOS study group has proposed diagnostic criteria in 2013 and 2016, such as symptoms present for at least 12 weeks, extending beyond the distribution of a single cervical nerve root or peripheral nerve and, that had not been satisfactorily explained by another condition and met at least one criterion in at least four of five specific categories (Thompson et al., 2013; Thompson, 2021). In these criteria, only two provocative maneuvers have been retained: the upper limb tension test (ULTT) and the 3-min elevated arm stress test (EAST). These criteria still need to be consensually accepted and used in order to obtain homogeneous and comparable populations in further studies. Anterior scalene blocks have also been described as good criteria to confirm the diagnosis of NTOS due to muscular impingement, as their positivity (pain relief) seem to predict good surgical results in more than 90% of the cases (Jordan and Machleder, 1998; Rochlin et al., 2012). The problematic of differential diagnosis is particularly important in athletes as the prevalence of TOS is higher in their population than in the general population (cervical injuries, shoulder calf syndrome, and canalar syndromes) (Richardson, 1999; Wang and Crielaard, 2001; Mosley, 2003; Safran, 2004; Toth et al., 2005; Reeser, 2007; Povlsen and Povlsen, 2018; Zuckerman et al., 2019). Therefore the incidence of TOS is difficult to evaluate, probably about 3–80 cases per 100,000 inhabitants in the general population (Jones et al., 2019; Masocatto et al., 2019) with a mean age of 20–30 for vascular TOS and 30–40 for the N-TOS (Grunebach et al., 2015; Masocatto et al., 2019). TOS might have a female predominance, especially for N-TOS (Balderman et al., 2017; Jones et al., 2019; Masocatto et al., 2019). However, these epidemiological data do not seem concordant with the finding in groups of Athletes (cf. results). Indeed, sport is described to be an independent risk factor in the development of TOS, especially at high level (Shutze et al., 2017; Povlsen and Povlsen, 2018). Recently, Ohman and Thompson proposed specific guidelines about TOS diagnosis and management in overhead athletes (Ohman and Thompson, 2020). However, these guidelines were based on low quality and most of the time unspecific studies with incomplete information concerning epidemiology and holistic management. The specific context of high-level athletes is a big part of the TOS problematic and requires a dedicated literature to answer specific questions such as epidemiology, specificities of management, sport performance and return to competition. The aim of this systematic review was to evaluate the scientific literature available for the management of TOS in the specific population of athletes because of a potential important socio-economic impact, especially in professional and high-level athletes due to the risk of anticipated sports season endings but also early career endings.

## Materials and Methods

### Search Strategy and Study Selection

To conduct this review, the PRISMA method was applied (Page et al., 2021). We searched in various databases (PubMed, Medline, Google scholar) the following key words:

- « Thoracic outlet syndrome » + « Sport » or « Physical Activity » or « Athletes ».
- « Paget Schroetter syndrome » + « Effort Thrombosis » + « Physical Activity » or « Sport » or « Athletes ».

Article research extended to March 2021 without other restriction concerning the date of articles publication. The search was performed independently by two assessors (TG, AFC) to assess titles and abstracts of potentially relevant articles, and then the full-text articles were retrieved. A first preselection based on the article titles was produced, regarding their availability in English or French and a second preselection was produced after reading the abstracts. In case of doubt, a third assessor’s advice was asked (PD).

### Eligibility

Case reports were selected only if the sport involved was documented, as well as the level of practice and clear explanations about how the diagnosis was conducted and eventually information about the management. In case of uncertain diagnosis or lack of critical information, the case report was excluded. Cohorts were included if data about the number and the sport level of athletes were detailed. Concerning guidelines and review of literature, we included the most recent ones and those specifying involvement of sport or physical activity.

## Results

**FIGURE 1:**
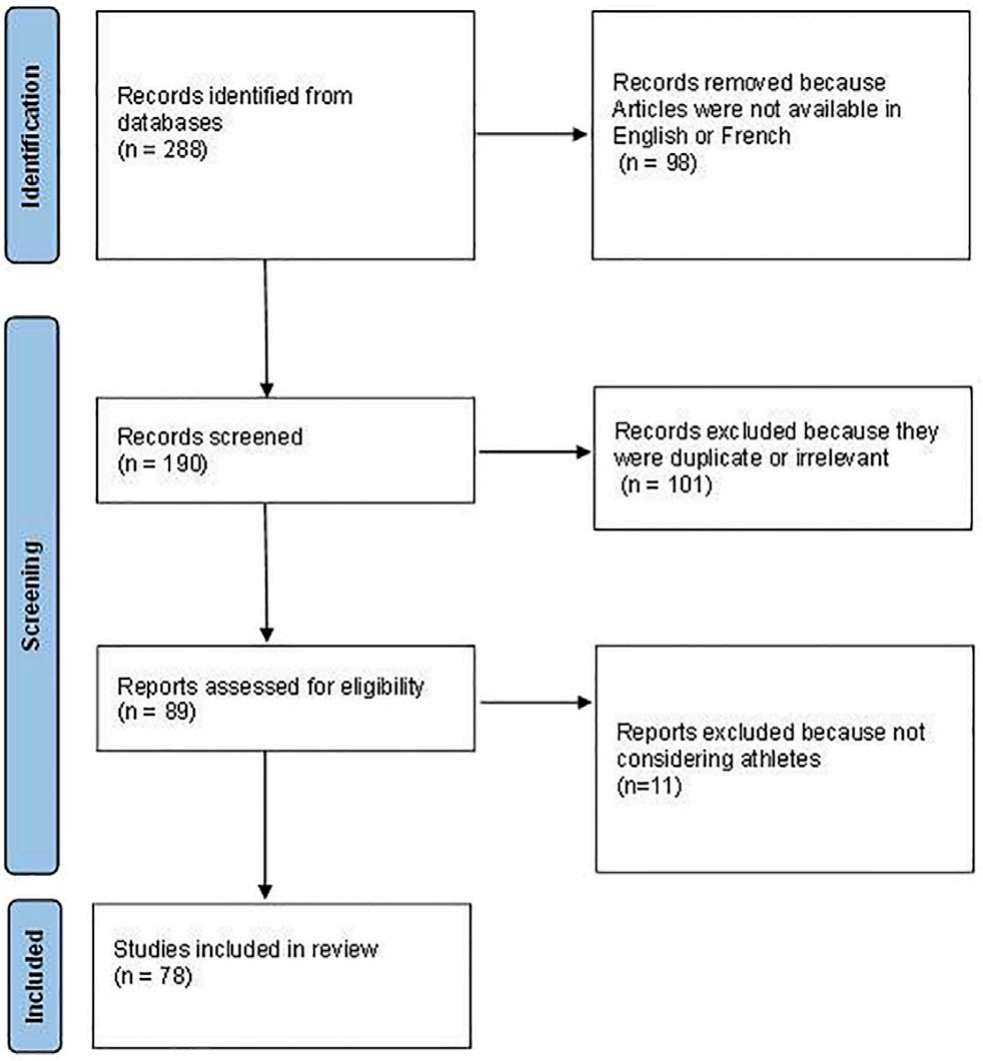
Flow chart of the process of article selection.

We selected 288 articles matching our criteria. The English or French abstract or the integral text was available for 190 of them. After process, 78 articles were selected for this review including 40 case reports, 10 clinical studies and 28 reviews of literature. Flow-chart is detailed in Figure 1. By grouping all these 78 articles, we attempted to answer some pragmatic questions concerning the diagnosis and the management of TOS in the specific population of athletes.

### Which Sports are at Risk of Thoracic Outlet Syndrome Development?

As previously mentioned, the essential of the scientific literature regarding TOS in athletes is mostly composed by surgical series and case reports. Sports involving upper arm strength and repetitive overhead motion seem to be the most described in TOS literature. Indeed, most case reports concern baseball pitchers and swimmers even if TOS is reported in several other sports such as softball, weightlifting, water polo, football (United States), lacrosse, archery, and athletics (cf next chapter: *What are the identified risk factors of TOS in Athletes?*). The involvement of these particular sports in TOS is supported by several series of athletes such as a series of 41 athletes treated in the United States including 13 baseball players, 13 swimmers, 5 water polo players, four rowers and two volleyball players (Chandra et al., 2014). Most of the case reports involve also baseball pitchers and swimmers (Wang and Crielaard, 2001; Richardson, 1999; Shutze et al., 2017; Yagi et al., 2017; VanWye et al., 2016; Thompson et al., 2017; Norinsky et al., 2016; Zajac et al., 2013; Herbst and Miller, 2013; Nitz and Nitz, 2013; Bushnell et al., 2009; Almeida et al., 2007; Hurley et al., 2006; McMaster, 1996; Katirji and Hardy, 1995; Fields et al., 1986; Vogel and Jensen, 1985; J. Gutman et al., 2021). Another series of 97 athletes diagnosed with TOS, also included 47 baseball or softball players, 11 volleyball players, eight gymnasts and surprisingly only two swimmers (Beteck et al., 2019). However, the main difference between the two series is that the first one included N-TOS and V-TOS and the second one exclusively N-TOS.

Another interesting question is to know if athletes involved in any sport are at higher risk of developing TOS. It seems that strength training (which is required in many sport preparation) is by itself a risk factor of TOS (Basile et al., 2001; Roche-Nagle et al., 2007; Kellar and Trigger, 2014; Jourdain et al., 2016; DeLisa et al., 2017; Umana et al., 2019). This hypothesis is also supported by several case reports reporting TOS in several other sports with sometimes less involvement of overhead arm motion such as soccer (Zakaria et al., 2020), cycling (Smith et al., 2008), judo (Ijaopo et al., 2016), running (Leung et al., 1999), climbing (Lutter et al., 2015) or triathlon (Sancho-González et al., 2017). However, TOS is a rare condition, and its diagnosis implies the elimination of many and more frequent differential diagnoses and it also implies the evaluation of the existence of other risk factors such as coagulation disorder, predisposal anatomical factor or muscle hypertrophy. Besides, sport related trauma can directly or indirectly lead to the development of TOS such as collarbone fractures, rib fractures or the development of bone callus reducing the size of one or more thoracic outlet area (Tucker, 1994; Casbas et al., 2005; Terabayashi et al., 2010; Nichols and Seiger, 2013; Kirby et al., 2015; Puttmann et al., 2016; VanWye et al., 2016; Funakoshi et al., 2019; Funakoshi et al., 2019; Zuckerman et al., 2019). For example, bone fractures are common in the history of Paget-Schroetter syndrome (Illig and Doyle, 2010).

### What are the Identified Risk Factors of Thoracic Outlet Syndrome in Athletes?

Several pathophysiological hypotheses have been detailed regarding the general population or the athletic population. Risk factors can be divided into intrinsic and extrinsic ones.

Intrinsic factors usually include anatomical considerations. Indeed, athletes combine nonspecific factors (such as cervical rib or coagulation anomaly) and more specific factors such as muscle hypertrophy, especially of the pectoralis minor, inducing the compression of the adjacent neurovascular bundle. This hypothesis is sustained by the frequent observation of muscle hypertrophy during TOS surgical procedure (Katirji and Hardy, 1995; Ozcakar et al., 2005; Simovitch et al., 2006; Almeida et al., 2007; Aydoğ et al., 2007; Baltopoulos et al., 2008; Fitzgerald, 2012; Herbst and Miller, 2013; Jourdain et al., 2016; Norinsky et al., 2016; Ciampi et al., 2017). Bottros et al. (2017) also proposed a diagnostic test of N-TOS based on ultra-sound guided scalenus anticus anesthesia. Muscle hypertrophy was described in two cases of N-TOS prior to the anesthesia using ultrasound imaging and the test was positive, relieving the patient of their symptoms in exercise conditions. Local anesthesia can inhibit muscle contraction of the scalenus anticus, leading to the decrease of the dynamic compression of the brachial plexus during exercise. Several other studies have reported similar findings in complex TOS diagnosis (Bottros et al., 2017).

The prevalence of cervical rib is estimated at 1% in the general population (Henry et al., 2018). However, it is difficult to know if this proportion is accurate in the specific population of athletes. Indeed, TOS symptoms might have discouraged at a relatively young age the pursuit of high demanding upper arm involvement required in many sports. It is interesting to underline that cervical rib is strongly associated with TOS, as it is found in half of the N- and V-TOS population according to Henry et al.’s meta-analysis (Henry et al., 2018). This contrasts with the finding of Illig and Doyle (2010) suggesting that cervical rib was not associated with the development of V-TOS. Regarding the extremely rare A-TOS condition, in the pediatric series of Matos et al. (2018) four of the eight patients diagnosed with A-TOS presented a cervical rib. Unfortunately, in most of the other series of cases the presence or the absence of cervical rib is not specified. Only a few cases reported the involvement of cervical rib in athletes (Craig and Knepper, 1937; Karageanes and Jacobs, 1998; Casey et al., 2003).

Concerning the other intrinsic risk factors described in the literature, the sex factor is debated in the athlete population (Shutze et al., 2017; Matos et al., 2018; Beteck et al., 2019; Jones et al., 2019). The presence of a coagulation disease is still associated with vascular TOS, especially PSS (Carpenetti and Grosel, 2018; Matos et al., 2018).

Otherwise, extrinsic factors are more linked to sport practice itself or training. In fact, in the field of strength training, several techniques have been incriminated as potential risk factors of TOS development (especially PSS) such as blood flow restriction or Kaatsu training (Noto et al., 2017). Yet, a systematic review was unable to conclude, certainly due to the rarity of the event (Minniti et al., 2019).

Baseball seems to be the most incriminated sport in the literature, particularly the pitchers, especially in the development of A-TOS (Duwayri et al., 2011; Zajac et al., 2013). Significant arterial blood flow drop was observed in pitchers with shoulder laxity compared to the same population without shoulder laxity. This could be explained by chronic compression of the axillary artery with the anterior translation of the humeral head during the throwing phase. In the long term, this can induce aneurysm of the arterial wall, dysplasia and even arterial blood clots. Lesions of the axillary artery are observed especially in the context of cervical rib (Duwayri et al., 2011; Zajac et al., 2013). These findings are important due to the rarity of the condition, which makes every case very valuable.

Regarding V-TOS, 60%–80% of the patients treated for PSS reported high intensity physical training involving upper arms within the few days prior to the event (Illig and Doyle, 2010). Therefore, it seems that high intensity sport training could lead directly to PSS - probably in predisposed patients (Illig and Doyle, 2010). Some authors suggested that repetitive motion could produce chronic inflammation of the vessels wall inducing hyperplasia and restraining blood flow, leading finally to clots and thrombosis (Illig and Doyle, 2010; Menon et al., 2019).

Finally, the originality of athletes with TOS is their young age compared to the general population, suggesting a direct link between upper arm over solicitation and the development or the acceleration of TOS symptoms in predisposed patients (Chandra et al., 2014; Matos et al., 2018; Beteck et al., 2019).

### What is the Management of Thoracic Outlet Syndrome in Athletes?

In TOS literature, surgical management is described to be performed after failure of conservative management, especially for N-TOS and seems to be reserved for the most severe forms (Jones et al., 2019). However, vascular TOS seem to benefit frequently from surgical procedure (Urschel and Razzuk, 1998). Regarding PSS, first intention surgery involves trans-axillary first rib removal with excellent reported outcomes (Urschel and Razzuk, 1998; Illig and Doyle, 2010). Anticoagulant therapy is prescribed for 3–6 months. Early thrombolysis, perioperative balloon venoplasty and early thoracic outlet decompression by first rib excision may provide better outcomes in V-TOS, especially to limit recurrent thrombosis with deferred decompression (Bamford et al., 2012; Madden et al., 2019; Ohman and Thompson, 2020). Several series of cases could help understand the management of TOS in athletes. Indeed, it has been confirmed by experience-based studies and reviews (DiFelice et al., 2002; Farrar et al., 2014). In the literature on general TOS population management, surgical approaches usually depend on the surgeons’ habits or preferences, and several surgeries have been described such as transaxillary, supraclavicular, or paraclavicular approaches. They seems to be used with rather good results in the surgical series in the three types of TOS (Urschel, 1996; Desai et al., 2014; Siracuse et al., 2015). In V-TOS, the infraclavicular approach seems to be a safe alternative and has good outcomes, with low complication risks compared to other surgical strategies (Siracuse et al., 2015; Madden et al., 2019). Yet, recurrences may be rather related to missteps during the initial operation than linked to the surgical approach (Archie et al., 2018).

Matos et al. (2018) reported in a surgical series of 68 pediatric patients (mean age of 15 years-old) a predominance of vascular TOS (V-TOS in 57% of cases and A-TOS in 12% of cases), which is not the usual epidemiology of TOS in the general population. Sport was the first risk factor found in 34% of the cases and up to 40% of the cases in V-TOS. As expected, a coagulation disease was also found in 22% of the cases. Regarding management, each patient with N-TOS benefited from a 6 month-physical therapy program before surgery (which might be challenging in the context of athletes pressured to return to competition). They reported excellent outcomes after surgery with 90% of the patients returning to their prior level of practice within a year.

Chang et al. (2009) also reported a 2-year-follow-up retrospective series of 70 patients after surgery. This follow-up concerned a general population (mean age from 35 to 40 years-old). We do not have much information concerning sport or physical activity in this study, but it confirmed that patients treated for V-TOS were younger than patients with N-TOS and had better outcomes with less time of physical therapy compared to N-TOS surgery (2 vs. 4 months). Regarding the functional outcomes, prognosis was also better in vascular forms with 77% of patients returning to work during the follow-up vs. 50% in N-TOS. Duration of symptoms prior to surgery was also longer in N-TOS (36 months) than in V-TOS (4.5 months). This underlines the extreme difficulty to recognize and diagnose properly N-TOS due to the presence of multiple co-factors and differential diagnoses, while vascular TOS diagnosis is more consensual. The authors underlined that 27% of the N-TOS patients had previously undergone a surgery due to their symptoms (cervical spine, elbow, and shoulder) and that only 45% of them benefited from a TOS specific rehabilitation.

Rinehardt et al. (2017) reported a retrospective series of 1,431 patients (selected in multiple TOS centers in the United States) who underwent TOS surgery. As previously observed, the epidemiology of theses surgical series differs from the usual epidemiology of TOS with 83% of N-TOS, 12% of V-TOS and 3% of A-TOS, because vascular TOS almost systematically benefits from surgery. Unlike precedent series, the A-TOS population seemed to be older than the mean population (45 vs. 34 years old). This study gives us some interesting data regarding the safety of surgical procedures. More complications (readmissions) were observed after V-TOS surgeries (15.4%) than for A-TOS (10.5%) and N-TOS (7.85%) surgeries.

Finally, Lugo et al. (2015) compared outcomes regarding PSS management over more than 500 patients in a literature review. They reported better outcomes after surgical removal of the first rib, with 93%–95% of clinical improvement, than after conservative treatment with thrombolysis, with only 54% of clinical improvement. However, the authors did not specify the duration of the follow-up and the protocols and if the procedures were standardized.

In the recent guidelines published by Ohman and Thompson (2020) for TOS in overhead athletes (i.e., athletes that use repetitive movements of the arm overhead), surgery is recommended for every patient. The supraclavicular approach might be preferred to the trans-axillary approach in N-TOS with immediate post-surgical rehabilitation for 9–12 months. V-TOS thrombolysis was not considered as a long-term treatment and anticoagulation should be prescribed after surgery for about 3 months.

Botulinum toxin has also been proposed in the management of patients with TOS but has never been specifically assessed in athletes. To our knowledge, only one study has evaluated its interest with high methodology in a randomized, double-blind controlled trial but it has not shown significant symptoms improvement (Finlayson et al., 2011).

### Do Outcomes Differ Between Athletes and General Population?

Beteck et al. (2019) compared athletes who underwent N-TOS surgery to non-athletes from 2009 to 2014. All patients had first rib resection associated to scalenectomy. They assessed retrospectively 184 patients including 97 athletes. The first observation was that athletes were younger than the other patients (mean age of 23 vs. 38 years old). Athletes used also fewer analgesic medications after surgery (61% vs. 80%) and were more likely to declare themselves cured (40% vs. 28%). In the same way, 77% of the athletes declared no limitation for *activities of daily living* vs. 56% for the non-athletes. These data suggest that athletes may have better prognosis than non-athletes after N-TOS surgery. This point was confirmed by multivariate analysis adjusted on age, gender, and rehabilitation. However, the athletes group was heterogeneous, containing patients with various level of physical activity and also soldiers and musicians (Beteck et al., 2019). Another issue is that the proportion of athletes who answered to this ancillary follow-up study might not be representative of the entire cohort. Indeed, in another article using the same cohort of 564 patients (Shutze et al., 2017), only 221 were athletes, which represents above 40% of the whole population compared to the 53% of athletes in the other article (Beteck et al., 2019). This might induce an over representation bias. Similarly, we do not know the proportion of athletes who did not undergo surgery to estimate the proportion of surgical management in athletes.

Chandra et al. (2011) also reported the outcomes of a prospective study (but compared to a retrospective cohort) concerning the evolution of patients 1 year after N-TOS surgery. The aim of the study was to improve the selection of patients for surgery. The prospective part of the study consisted in selecting only patients who had undergone a TOS specific physical therapy (not detailed). Only the patients who had benefited from this physical therapy were selected for surgery. The comparison seemed difficult between the prospectively selected group after physiotherapy and the retrospectively selected one, but the authors reported better outcomes after applying these selection criteria. In a way, this physical therapy may be seen more as a diagnostic criterion since it may have helped discriminate N-TOS from differential diagnosis. Yet, in the last guidelines by Ohman and Thompson (2020), this algorithm is no longer recommended since it has failed to improve surgical outcomes as demonstrated in a meta-analysis.

### What is the Prognosis for the Return to Competition?

Chandra et al., (2014) reported the outcome of all the athletes (from high school to professional leagues) treated in their center between 2000 and 2012 for TOS. This team, specializing in vascular surgery, reported 41 athletes treated for N-TOS or V-TOS. All the V-TOS patients benefited from a first rib ablation with anticoagulant treatment and 93% returned to prior level of sport. The N-TOS group was more heterogeneous because the authors modified the selection of their patients in 2007 using an algorithm based on improvement after TOS specific physiotherapy. However, 81% of the pooled N-TOS population was able to return to previous level of physical activity. The authors underlined that only seven of their patients, who had not undergone surgery (recused by their algorithm), were able to return to their previous sport level. The meantime to return to sport was 4.7 months and the recidivism rate was 7% for N-TOS and 14% for V-TOS.

Beteck et al. (2019) and Shutze et al. (2017) also described above 70% of their athletes able to return to previous level of physical activity with about 50% within a year. However, 37% of their patients underwent a second surgical procedure. Young age was associated with better and faster recovery.

Farrar et al. (2014) presented in a review of literature outcomes after PSS. While they exposed few data about athletes, a consensus of return to physical training after 12 weeks was proposed after the anticoagulant treatment ending. Another study suggested that adapted program of physical activity was possible under anticoagulant treatment (DiFelice et al., 2002). Finally, Melby et al. (2008) proposed a program of immediate and progressive rehabilitation after surgery. Athletes returned to activity at 3.5 months (3–10 months).

Thompson et al. (2017) specifically designed a study to answer the question of the return to competition after TOS surgery. They reported the outcome of 13 Major League Baseball (MLB) players that had undergone TOS surgery. Ten patients were able to return to competition, 1 year after surgery and six of them continued to play in a professional league for at least 3 years. The authors also reported that the other four patients did not end their career in relation with TOS. Statistical analysis of MLB demonstrated that these 10 players did not decrease their performances on field according to 15 different sports parameters. More recently, Gutman et al. (J. Gutman et al., 2021) assessed 27 MLB pitchers who underwent surgical treatment for TOS (20 NTOS and seven VTOS). Twenty of them (74%) were able to return to play in MLB at mean delay of 297 days (14 NTOS and six VTOS). The majority of MLB metrics demonstrated that these players returned at a similar level of sport.

The TOS literature lacks specific studies on athletes, particularly about non-surgical management. However, some studies detailed the importance of physical therapy in first intention even if we are unable to evaluate the proportion of surgery in this specific population (Huang and Zager, 2004; Levine and Rigby, 2018).

## Perspective and Conclusion

TOS is still poorly understood especially N-TOS, due to the lack of strong diagnostic criteria and exams, poor specificity and high variability of symptoms with many differential diagnoses (Fouasson-Chailloux et al., 2021b). This difficulty increases because of the low frequency of TOS, making it difficult to recognize and inducing delay of management (Dragu et al., 2009). TOS is also a constructed entity, grouping heterogeneous manifestations, which need specific treatments according to the type and the localization of the compression, the pathophysiology and the patient expectation. Then, it seems necessary to address specific studies for each type of situation, especially for the athletes who are particularly concerned.

Despite a recent proposition of diagnostic criteria for N-TOS (Thompson et al., 2013), further studies are needed. Indeed, as underlined by Povlsen et al. (2014), the therapeutic studies at our disposition lack reliable methodology necessary to construct guidelines. We can only rely on the experience of several case reports (mostly surgical) to have an overview of the actual management and outcomes.

Athletes seem to represent a high proportion of TOS patients with a distinctive epidemiology (younger age and more vascular forms) and specific problems to address, which deserves a more specific literature. Indeed, athletes are highly pressured patients with high expectations regarding return to competition and delay of recovery. However, these high expectations and pressure must not lead to excess of treatment that could be detrimental to the patients in the long term. To our knowledge, there is no long-term follow-up study of athletes after surgery nor comparison of long-term outcomes between surgery and conservative management. It is also important to improve diagnostic quality to recognize early symptoms and to eliminate differential diagnoses. Another issue is the definition of what a high-level athlete is. Indeed, there is no consensus regarding the definition of high-level athletes and it is poorly described in studies. Yet, they are usually assimilated to professional and national/international sports people (Shutze et al., 2017).

To summarize, this review of literature underlines an increased risk of developing TOS in the context of sport practice, especially sports involving upper arm with repetitive movements, such as swimming and pitching in baseball. In these particular sports, medical staff is certainly familiar with TOS leading to high rate of diagnoses and early management. When pooled, all the experience-based studies seem to demonstrate favorable outcomes for athletes after surgical management in the 1–4 years after treatment, but with a significant rate of complications and relapses (from 8% to 15% of complications) (Rinehardt et al., 2017). Furthermore, more data about conservative management are needed. It consists in physiotherapy protocols that are unfortunately not standardized and still poorly described (Vanti et al., 2007). However, the management of severe vascular TOS seems more consensual despite the absence of guidelines. Vascular TOS prevalence increases in athletes, and surgery is performed in first intention. The specific question of the anticoagulation treatment in the context of athlete’s rehabilitation must also be addressed. TOS is still a challenging topic in the matter of diagnosis and management as it is rare and probably at the same time under and over diagnosed with potential severe complications especially in vascular forms and a high variety of symptoms. Most of the management seems to involve surgical procedures despite a lack of controlled studies and validated therapeutic protocols. The validation of this experience-based management by good quality studies seems necessary.
